# Assessment of genetic variation in *Apis mellifera jemenitica* (Hymenoptera: Apidae) using Cytochrome Oxidase I gene sequences

**DOI:** 10.1016/j.sjbs.2021.07.033

**Published:** 2021-07-16

**Authors:** A. Alghamdi, Yehya Alattal

**Affiliations:** Abdullah Bagshan Chair for Bee Research, Department of Plant Protection, College of Food and Agricultural Sciences, King Saud University, P.O. Box 2460, Riyadh 11451, Saudi Arabia

**Keywords:** Arabian Honeybee, Saudi Arabia, Genetic variation, *Mt*DNA, *Apis mellifera jemenitica*

## Abstract

The Arabian Honeybee *Apis mellifera jemenitica* is endemic to the Arabian Peninsula. It is highly adapted to temperature extremes and drought dominating the region. In this study, the mitochondrial Cytochrome Oxidase I (COI) was analyzed in 133 specimens of A. m. jemenitica from eight localities along the Red Sea cost of Saudi Arabia. Results revealed 33 synonymous, and 6 non-synonymous mutations within the COI sequences, resulting in change of 4 amino acids. Phylogenetic analysis based on either type of mutations revealed two main haplogroups accounting for 94% of the samples. In total Eighteen new haplotypes were identified and uploaded in the genebank, Fourteen of them are restricted to one/both haplogroups. All haplotypes identified in this study clustered with reference COI sequences of the sub-lineag Z (African Lineage). However one Haplotype (MW428270) represents high COI variability compared to other haplotypes and may resemble different evolutionary sub-lineage. Tajima's Neutrality Test (Ps = 0.025; D = -1.5) indicated population size expansion that took place after selective sweep and/or purifying selection.

## 1 Introduction

Both morphometric and genetic analyses are basically used to characterize honeybee subspecies and ecotypes (Ruttner, 1988, Garnery et al., 1992, Meixner et al., 2013, Henriques et al., 2018). Based on morphometric approaches, 33 subspecies were identified and assigned to four different lineages (African (A); Western Europe (M); South-Eastern Europe (C) and Middle East (O)) demonstrating high consistency with genetic approaches based on *mt*DNA markers (revised by Ilyasov et al., 2020). The mitogenomes evolve more rapid and may provide earlier diagnostic markers compared to nuclear markers (Moore, 1995, Kaltenpoth et al., 2012). The Arabian Honeybee *Apis mellifera jemenitica* is the endemic honeybee of the Arabian Peninsula. It spreads naturally in Africa and Asia and is highly adapted to environmental extremes (Ruttner, 1988, Alattal and Alghamdi, 2015). In Saudi Arabia it is mostly managed in the southern and western regions parallel to the Red Sea coast along the Siriwat mountain series of Saudi Arabia. The endemic honeybee of Saudi Arabia is a member of the African sub-lineage Z (Previously O lineage) (Alburaki et al., 2013, Alattal et al., 2014). However in a recent phylogenetic study (based on PCGs phylogenetic analysis of 14 *A. m. jemenitica* mitogenomes from Saudi Arabia) mitogenome clustered into three sub-groups, two of them are considerably different, which may indicate a membership of two different lineages. A problem in the use of such phylogenetic tree is the limited information on the variability of mitogemones sequences within other *A. mellifera subspecies*. For sequence alignment purposes, most honeybee subspecies are represented by one mitogenom in the genebank (NCBI), however much data is available when only one mitochondrial gene or region is considered for construction of phylogenetic trees and investigation of relationships among honeybee subspecies or lineages. In this study 133 sequences of the COI gene of the *A. m. jemenitica* from Saudi Arabia were aligned with other sequences of honeybee subspecies previously reported in the genebanck (NCBI). Our main target is to investigate the amount of variation in COI gene within these sequences and to use them in diagnosing and illustrating their phylogenetic relationship with other honeybee subspecies.

## 2 Materials and methods

**Fig. 1:**
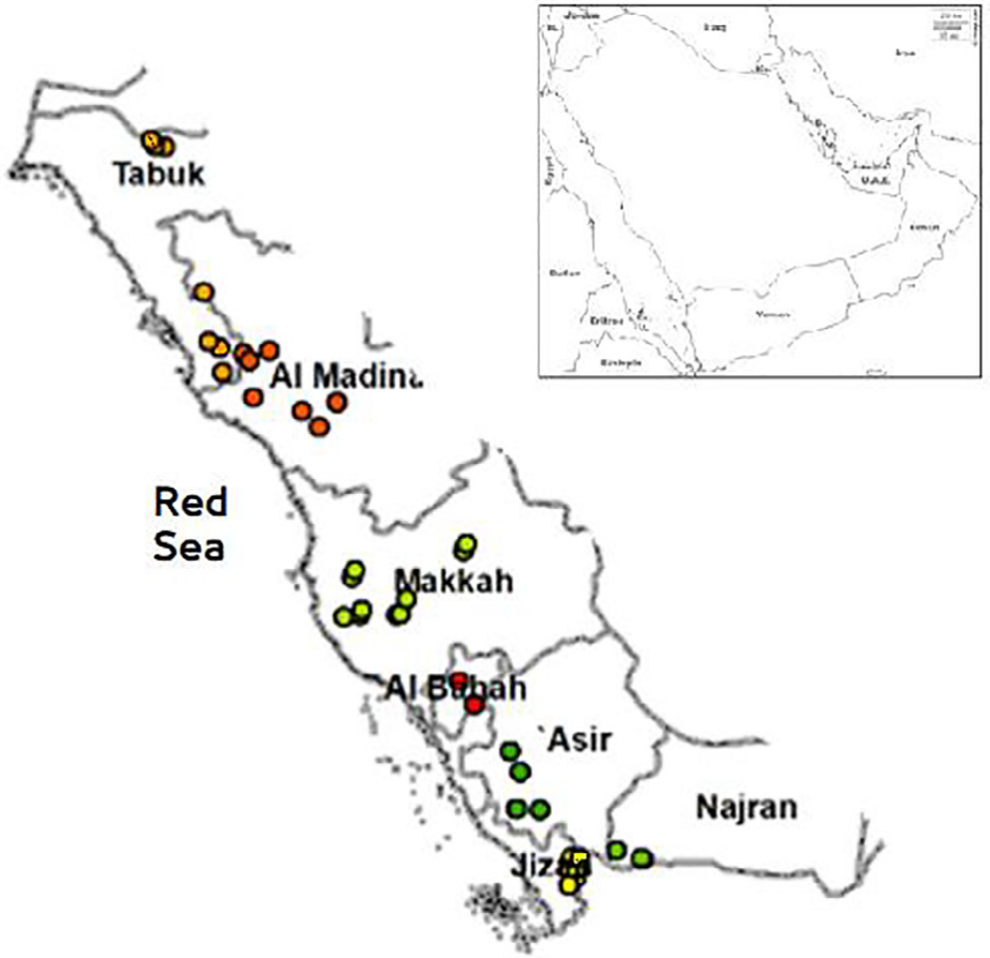
Sites of sampled apiaries along the Red Sea coast of Saudi Arabia; Tabuk (n = 18): 28.387028,36.855194; AlMadina (n = 17) 25.411561,37.520384; Makkah(n = 20): 21.911416,39.753706; Taif (14): 21.472722,40.551694; Albaha (n = 14): 19.852472,41.585611; Asir (19): 18.256722,42.229028 Jazan (n = 16) 17.517472,43.075000; Najran (n = 14) : 17.625917,43.754611.

Honey bee samples from 133 non-migratory *Apis mellifera jemenitica* colonies spread along the Red Sea cost of Saudi Arabia were collected (Fig. 1) and were then preserved in 96% Ethanol. Each sample consisted of 15 workers. Ten workers from each sample were dissected, body parts were mounted on slides, and scanned using a high resolution scanner (600 dpi) connected to desk-top computer system supported with image tool software (Image tool® 3.0). Twenty nine body characters (Table 1) were measured (Goetze, 1964, Ruttner, 1988). Colony means were then calculated for each character. Afterwards, reference data representing the measurements of the corresponding characters for 4 other *A. mellifera* subspecies from six countries (Syria, Egypt, Sudan, Uganda, Somalia, Italy) obtained from Oberursel Bee Research Institute (Frankfurt, Germany) were included in the data set. Subsequently, discriminant analysis using Wilk’s lambda was used to verify reallocation probabilities among different subspecies. Analysis was performed using Past 4.03 (Hammer et al., 2021). For *mt*DNA analyses, DNA was extracted from one worker bee per colony using Qiagen extraction Kit (Cat No./ID: 69506). Extracted DNA was then sequenced by BGI (Hong Kong, China). Raw data processing was performed using SOAPnuke v1.5.6 (parameters -n 0.05 -l 20 -q 0.2 –G –Q 2) (Chen et al., 2018). Filtration included three-step, started with adaptor trimming, any reads with adaptor mapping rate higher than 50% was removed. Then, low quality reads with more than 50% of low quality bases (Q20 < 50%) were removed. Finally, contiguous reads with more than 2 % N bases were removed. Trimmed mtDNA reads were mapped and annotated in Geneious Prime 2020.1.2 using a reference mitogenome of *A. m. jemenitica* (GeneBank: MN714161). Sequences of COI and for each sample were then extracted in fasta format and were aligned with mtDNA sequences of other *A. mellifera* subspecis using BioEdit v7.2.5 (Hall, 1999) and were subjected to restriction enzymes; TaqI and HinfI. Phylogenetic tree was constructed using Maximum Composite Likelihood method and tested over1000 bootstrap replicates (Felsenstein 1985), evolutionary distances as the number of base substitutions per site were calculated in MEGA7 (Kumar et al. 2016). Impact of nucleotide polymorphism on amino acid changes was also explored. Sequences were additionally analyzed with Basic Local Alignment Search Tool (BLAST) search program (National Center for Biotechnology Information site - NCBI), and compared with other sequences available in GenBank, then new haplotypes (haplotypes) were uploaded into the genbank.

**Table 1:** List of morphometric characters used in this analysis and their numbers as given by Ruttner (1988).

**Character**	**No.**	**Character**	**No.**
Length of proboscis	*4*	Pigmentation of tergite 3	*33*
Length of Femur	*5*	Pigmentation of tergite 4	*34*
Length of tibia	*6*	Wing Angel (a4)	21
Length of metatarsus	*7*	Wing Angel (b4)	22
Width of metatarsus	*8*	Wing Angel (d7)	23
LW_MTAR	*7:8*	Index of slenderness	*19:20*
Length of hind leg	*5 + 6 + 7*	Wing Angel (e9)	24
Tergite 3 longitudinal	*9*	Wing Angel (g18)	25
Tergite 4 longitudinal	*10*	Wing Angel (j10)	26
Forewing Length	*17*	Wing Angel (j16)	27
Forewing Width	*18*	Wing Angel (k19)	28
LWFW	*17:18*	Wing Angel (l13)	29
Cubital 1	*19*	Wing Angel (n23)	30
Cubital 2	*20*	Wing Angel (o26)	31
Pigmentation of tergite 2	*32*		

## 3 Results

**Fig. 2:**
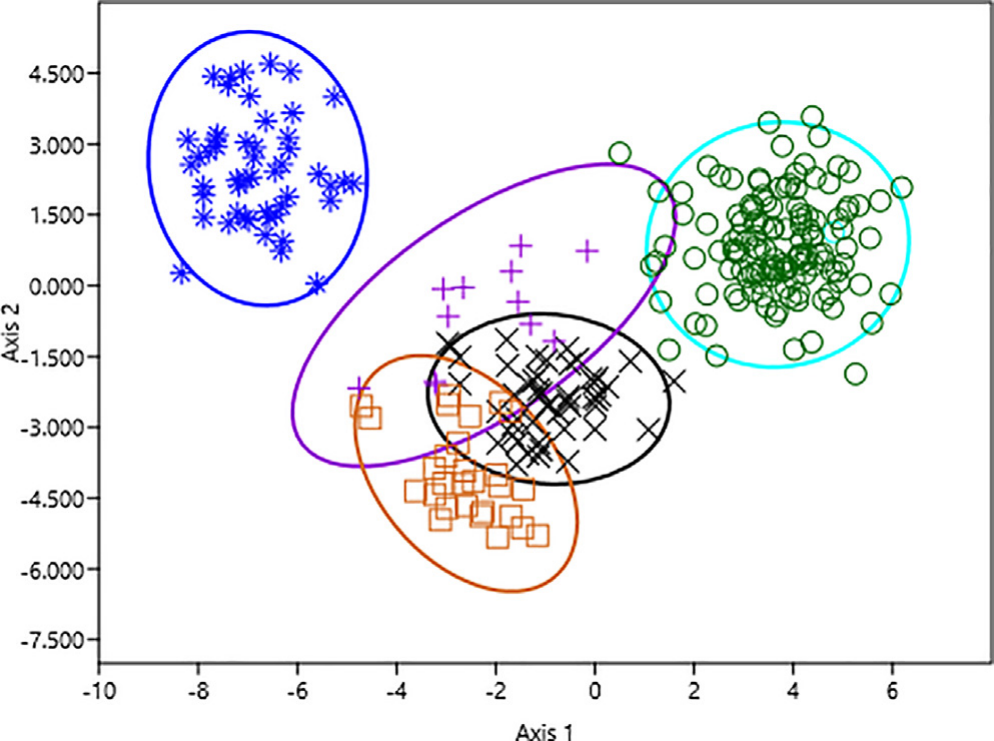
Discriminant analysis of colonies based on morphometrical data sets . the figure shows the colony means of trais of the Arabian Honeybee *A. m. jemenitica* samples from Saudi Arabia (o) and and data of four reference subspecies (*A. m. ligustica* n = 50 (*); *A. m. syriaca* n = 12 (+); *A. m. jemenitica* (African reference colonies) = 50 (×) and *A. m lamarckii* n = 28(□)) obtained from the state institue for beekeeping in Frankfurt (Prof. Stefan Fuchs).

**Fig. 3:**
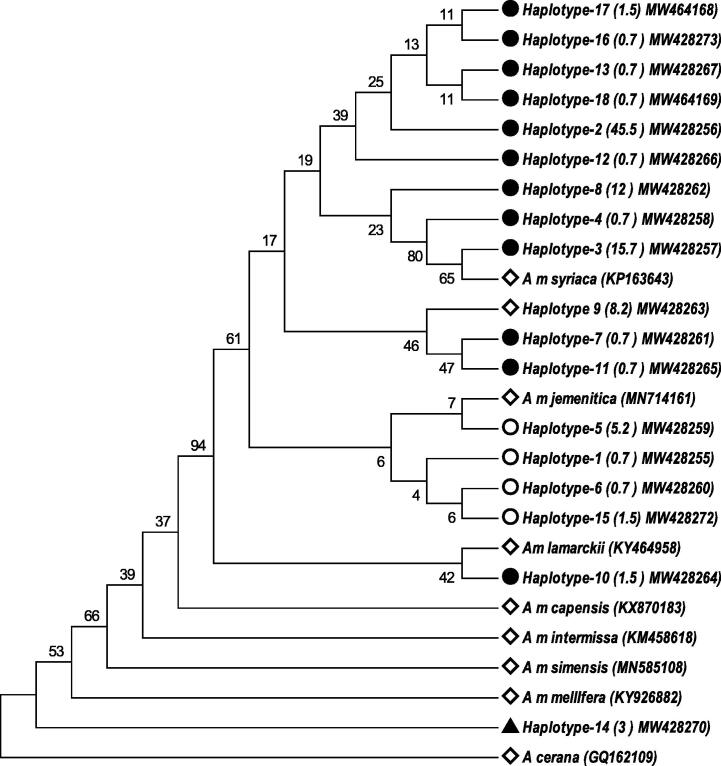
Maximum likelihood phylogenetic tree representing 18 *A. m.* *jemenitica* haplotypes using COI gene sequences. The number represents the bootstrap values which are shown behind each node. There were a total of 1572 positions in the final dataset. Evolutionary analyses were conducted in MEGA6.

Discriminant analysis of reference subspecies confirmed their allocation to their original groups (N = 140). However, in cross-validating grouping two colonies of the study samples (~1.5%) was allocated with the African *A. m. jemenitica* reference group, and one data set from each of *A. m. syriaca*, *A. m. lamarckii* and *A. m. ligustica* grouped unsuccessfully (Table 2) and were removed from further analysis. This analysis aimed to confirm subspecies identity of our samples. All of the Saudi samples clustered together and were closest to the reference *A. m. jemenitica* and *A. m. syriaca* (Squared Mahalabonis Distance = 29 and 37 respectively) (Fig. 2). The COI gene sequence length was 1572 bp and composed of 76.2 AT and 23.8 GC. Variable sites were 39 (2.5%) (Table 3) resulted in 6 variable amino acids (0.38%) (Table 4). Analysis revealed eighteen new haplotypes of COI gene sequences among *A. m. jemenitica* within Saudi Arabia (Table 5). One haplotype (haplotypes 14: n = 4 (~3%)) revealed the highest number of substituted nucleotides with one non-stop mutation (26n), and phylogenetically close to *A. m. mellifera* and *A. m. capensis* COI sequences. Other COI haplotypes are similar to *A. m. jemenitica*, *A. m. syriaca* and *A. m. lamarckii* reference sequences (Fig. 3). Digestion map of the COI region using *Taq1* revealed two different restriction patterns among our samples. COI haplotype 14 revealed four restriction sites (112, 286, 637, 904), while all other haplotypes had five restriction site (112, 286, 637, 660, 904) (Table 5). Digestion with Hinf1 revealed a different pattern for haplotype 14 as well, with two restriction sites (18, 1334), while all other haplotypes had 3 restriction sites (18, 883, 1334) (Table 5). All haplotypes were uploaded in the gene bank and their accession numbers are given in Table 3. Analysis of translated sequences of COI gene revealed four non-synonymous SNPs and demonstrated changes in three amino acids (Codon no. 128, 272, 521) among all haplotypes and one nonstop mutation in haplotype 14 (Table 4). Based on amino acid changes, most colonies clustered into two groups. The first two groups are common (77 and 48 colonies, respectively), the third group resemble haplotype 14 which demonstrated mutation in the termination codon.

**Table 2:** Grouping of the study samples and reference subspecies by cross-validating test.

	*A. m jemenitica* Saudi Samples	*A. m jemenitica* African Origin	*A. m. syriaca*	*A. m. lamarckii*	*A. m. ligustica*
*A. m jemenitica* Saudi Samples	129	2	0	0	0
*A. m jemenitica* African Origin	0	50	0	0	0
*A. m. syriaca*	0	0	11	1	0
*A. m. lamarckii*	0	0	0	27	1
*A. m. ligustica*	0	0	1	0	49
Total	129	52	12	28	50

**Table 3:** Sequence variations in COI gene among our honeybee samples and six other honey bee subspecies resembling two lineages. *The number of the nucleotide at the sequence resembles the position where variation took place.

**Table 4:** Amino acid variations in COI gene among different haplotypes and six other reference subspecies resembling two lineages. *The number of the codon resembles the position of variation.

**Haplogroup**	**Haplotype (distribution percent in the cluster)**	**No. of colonies**	**Codon No.**			**Termination codon**
			**128**	**272**	**521**	
			Amino acid symbol*			
I	(MW428260:100%); (MW428259:100%);(MW428261:100%); (MW428262:19%); (MW428263: 18%); (MW428264:100%); (MW428265:100%);(MW428267:100%); (MW428272:100%); (MW428273:100%); (MW464169:100%); (MW428256:100%)	**77**	**V**	**G**	**I**	
II	(MW428255:100%), (MW428262: 81%); (MW428263: 82%): (MW428257:100%)	**48**	**V**	**R**	**I**	
III	(MW428258:100%)	**1**	**I**	**R**	**I**	
IV	(MW464168:100%)	**2**	**V**	**G**	**T**	
V	(MW428266:100%)	**1**	**I**	**G**	**I**	
VI	(MW428270:100%)	**4**	**V**	**G**	**I**	**W**
*A m jemenitica (MN714161); A. m lamarckii (KY464958); A m intermissa (KM458618);* *A m capensis (KX870183)*			**V**	**G**	**I**	
*A m syriaca (KP163643); A m meda (KY464957), A m caucasica (MN714160.1)*			**V**	**G**	**R**	
*A m carnica (MN250878.1); A m ligustica (AP018435.1)*			**S**	**G**	**R**	
*A m mellIfera (KY926882)*			**V**	**I**	**I**	

* V = Valine; G = Glycine; I = Isoleucine; R = Arginine; S = Serine; T = Thereonine; W = Tryptophan.

**Table 5:** Haplotypes accession numbers, frequency within population and region and restriction map of different sequences using Taq1 and Hinf1.

COI	Accession No.	Freq. (%)	Distribution								Digestion fragment size							
											Taq1					Hinf1		
			Makkah	Madinah	Taif	Jazan	Najran	Tabuk	Albaha	Asir	I	II	III	IV	V	I	II	III
Haplotype			No. (%)								112	286	637	660	904	18	883	1334
1	MW428255	0.7	**–**	**1(1 0 0)**	**–**	**–**	**–**	**–**	**–**	**–**								
2	MW428256	45.5	14 (23)	4 (6.6)	4 (6.6)	9(14.8)	10(16.4)	5(8.2)	7(11.5)	8(13.1)								
3	W428257	15.7	1(4.8)	11(52.4)	–	–	1(4.8)	8(38.1)	–	–								
4	MW428258	0.7	–	1(1 0 0)	–	–	–	–	–	–								
5	W428259	5.2	2 (28.6)	–	1(14.3)	1(14.3)	–	–	–	3(42.9)								
6	W428260	0.7	–	–	–	–	–	–	–	1(1 0 0)								
7	MW428261	0.7	–	–	–	–	–	–	–	1(1 0 0)								
8	MW428262	11.9	2(12.5)	–	7(43.8)	1(6.3)	–	–	2(12.5)	4(25)								
9	MW428263	8.2	–	–	–	2(18.2)	–	4(36.4)	3(27.3)	2(18.2)								
10	MW428264	1.5	–	–	–	–	–	1(50)	1(50)	–								
11	MW428265	0.7	–	–	–	–	–	–	1(1 0 0)	–								
12	MW428266	0.7	–	–	–	–	–	–	1(1 0 0)	–								
13	W428267	0.7	–	–	–	1(1 0 0)	–	–	–	–								
14	W428270	3.0	–	–	–	2(50)	2(50)	–	–	–	112	286	637	904		18	1334	
15	MW428272	1.5	–	–	2(1 0 0)	–	0	–	–	–	112	286	637	660	904	18	883	1334
16	MW428273	0.7	–	–	–	–	1(1 0 0)	–	–	–								
17	MW464168	1.5	–	–	–	2(1 0 0)	–	–	–	–								
18	MW464169	0.7	1(1 0 0)	–	–	–	–	–	–	–								
*A. m jemenitica (*MN714161); *A. m syriaca (*KP163643*; A. m lamarckii (*KY464958)											112	286	637	660	904	18	883	1334
*A. m simensis (*MN585108); *A. m capensis (*KX870183)											637	660	904	904	–	18	1334	–
*A. m intermissa (*KM458618)											112	637	660	904	–	18	1334	–
*A. m mellifera*	KY926882										112	637	904	–	–	18	1334	–

## 4 Discussion

Although all samples demonstrated their identity based on morphometric analysis as *A. m. jemenitica*, they were distinct from their African population of the same subspecies; however it is not known how they are genetically different. This could be further investigated when more mitogenomes or COI sequences of the African *A. m. jemenitica* are available for comparison. Variation in COI sequences was higher in our samples (39 sites) compared with the analysis of 25 samples of *A. m. capensis* and *A m. scutellata* (Eimanifar et al., 2018) which revealed 34 sites among both African subspecies. Analyses using SNPs or the variation in amino acids (Nonsynonymous SNPs) of the COI clearly resulted in two main clusters with limited variation. Yet, Haplotype 14, which was confirmed morphometrically as *Apis mellifera jemenitica*, accounted for most of the variability in COI sequences of our samples with 26 variable sites and can be diagnosed by the occurrence of non-stop mutation within its COI gene sequences which may impact protein functionality, and should be further investigated. Results from Tajima's Neutrality Test indicated population size expansion that took place after selective sweep and/or purifying selection in the COI gene (*Ps* = 0.025; d = -1.6) within the study population. Phylogenetic tree of our samples (n = 133) using the Maximum Likelihood method demonstrated that some samples clustered very close to *A. m. mellifera* from C lineage and to other African subspecies revealing the same clustering pattern published by Alghamdi and Alattal (2020) based on complete mitogenome analysis of 14 Saudi Arabian *A. m. jemnitica*. This indicates that *A. m. jemenitica* from Saudi Arabia may resemble two different sub-lineages or lineages. However digestion by both restriction enzymes of the COI gene confirms that haplotype 14 is closer to African subspecies of the Lineage A compared with *A. m. melifera* from lineage C. Diagnosis based on amino acid sequence is also possible separate samples from north or south, for example Haplotypes of haplogroup II (MW428255), (MW428262: 81%); (MW428263: 82%): (MW428257)) are restricted to the northern regions of Saudi Arbia. Haplotype 14 (MW428270) is restricted to far south in Jazan and Najran as well as Haplotypes of haplogroup I. Ultimately, the high variability in COI gene sequences of Haplotype 14 compared to other samples within Saudi Arabia could be explained by several factors. First variation may represent hybrids of *A. m*. *jemenitica* (particularly if hybrids backcross to one of the parental) that are morphologically like *A. m. jemenitica*, but may retain the mitogenome of the second subspecies. But this may not be likely the reason as samples were restricted to non-migratory colonies and from apiaries which are protected from invasive honeybee subspecies. Second, there could be incomplete lineage sorting. Third, phenotypic differences among subspecies may be administrated by number of specific genes while the remainder of the nuclear genome (as well as the mitogenome) have not yet become reciprocally monophyletic (Frank et al., 2001). Nevertheless, the classification of the Saudi honey bee originating from this study could be used to group other bee populations in neighboring countries.

## 5 Conclusion

Most COI haplotypes of this study were identified as members of the sub-lineag Z (African Lineage). However four samples (3%) that resembles the same Haplotype (MW428270) represents high COI variability may a member of different evolutionary sub-lineage. We can conclude that honeybee samples of Saudi Arabia represent two sublineages based on COI Variants. Most COI haplotypes of this study were identified as members of the sub-lineag Z (African Lineage). However four samples (3%) that resembles the same Haplotype (MW428270) represents high COI variability may a member of different evolutionary sub-lineage. We can conclude that honeybee samples of Saudi Arabia represent two sublineages based on COI Variants.

## Declaration of Competing Interest

The authors declare that they have no known competing financial interests or personal relationships that could have appeared to influence the work reported in this paper.
